# Increased Autophagy Enhances the Resistance to Tumor Necrosis Factor-Alpha Treatment in Rheumatoid Arthritis Human Fibroblast-Like Synovial Cell

**DOI:** 10.1155/2018/4941027

**Published:** 2018-10-25

**Authors:** Yujie Dai, Jingjing Ding, Wen Yin, Yuzhu He, Fei Yu, Cong Ye, Shaoxian Hu, Yikai Yu

**Affiliations:** ^1^Department of Nephrology and Rheumatology, Puai Hospital, Tongji Medical College, Huazhong University of Science and Technology, Wuhan, Hubei, China; ^2^Department of Rheumatology and Immunology, Tongji Hospital, Tongji Medical College, Huazhong University of Science and Technology, Wuhan, Hubei, China

## Abstract

Tumor Necrosis Factor-alpha (TNF-*α*) was reported to increase autophagy in rheumatoid arthritis human fibroblast-like synovial cell (RA-HFLS). We investigated different levels of TNF-*α* exposed to RA-HFLS by focusing on the relationship of autophagy and apoptosis. RA-HFLS and normal human fibroblast-like synovial cell (HFLS) were stimulated by TNF-*α* in the presence or the absence of 3-methyladenine (3-MA) or chloroquine (CQ). Cell apoptosis was detected by flow cytometry. Autophagy was determined through the expression levels of LC3, Beclin1, and P62 measured by Western Blot analysis as well as Confocal Laser Scanning Microscopy. The basal autophagy level was significantly higher in RA-HFLS than in HFLS. Autophagy was enhanced both in RA-HFLS and HFLS when they were treated with TNF-*α*. With the treatment of TNF-*α*, a slightly higher autophagy level was found in RA-HFLS than in HFLS, without a dose dependent effect. When autophagy was inhibited by 3-MA or CQ, apoptosis increased in both groups. With the stimulation of different doses TNF-*α*, apoptosis was much higher in HFLS group than in RA-HFLS. Autophagy is a protection mechanism when treated by TNF-*α* in RA-HFLS.

## 1. Introduction

Rheumatoid arthritis (RA) is the most common autoimmune disease characterized by chronic inflammation and joint destruction [1, 2]. It is widely accepted that this inflammatory cell infiltration and destructive features in joint are driven by resident rheumatoid arthritis human fibroblast-like synovial cell (RA-HFLS). RA-HFLS produces large amounts of inflammatory cytokines, chemokines, and matrix-degrading enzymes, and these molecules thereby recruit and stimulate neutrophils, macrophages, and lymphocytes secreting inflammatory mediators to the synovial fluid, which further exacerbate the joint inflammation [3, 4]. Some evidence indicates that RA-HFLS appears to change its phenotype like tumor cells, which show invasive hyperplastic and lacking apoptosis [5]. Therefore, RA- HFLS plays a crucial role in the pathogenesis of RA.

Accumulating evidence indicates that autophagy appears to have a dual role in eukaryotic cells. It manifests cytoprotective effects through offering nutrient and the clearance of unnecessary materials and pathogens. However, extensive or persistent autophagy also contributes to cell death different from apoptosis pathway [6, 7]. Large amounts of evidence indicate that autophagy protects various cells from apoptosis [8, 9]. Compared with normal human fibroblast-like synovial cell (HFLS), increased induction of autophagy may be associated with resistance-apoptosis in RA-HFLS [10, 11].

Tumor necrosis factor alpha (TNF-*α*), as a common inflammatory cytokine, has been significantly increased in patients with RA. TNF-*α* level in serum and synovial fluid has a positive correlation with the disease activity of RA patients [12, 13]. Furthermore, TNF-*α* has been widely used as a therapeutic target in RA [14–16]. However, the influence and internal mechanism of different TNF-*α* levels regulating autophagy and apoptosis in RA-HFLS are still unclear.

Here, we describe different doses of TNF-*α* influenced autophagy in RA-HFLS and its relationship with apoptosis. On the basis of the present study, we report that TNF-*α* induced protective autophagy in RA-HFLS, but not in dose dependent manner. That enhanced autophagy protects RA-HFLS from apoptosis.

## 2. Materials and Methods

### 2.1. Cell Cultures and Treatment

RA-HFLS and HFLS, human synovial fibroblast cell line derived from patient with RA and normal human, respectively, were purchased from Jennio Biotech (Guang zhou, China). Cells were cultured as described elsewhere, in which cells were cultivated in DMEM supplemented with 10% fetal bovine serum and kept at 37°C in 5% CO2. The concentrations of TNF-*α* (Peprotech, USA) used in the different experiments ranged from 0 to 100ng/ml *μ*M, and these two synovial fibroblasts were continuously exposed to TNF-*α* for 48 h. Controls were treated with matched amounts of phosphate buffer solution (PBS). 3-MA was purchased from Sigma-Aldrich (St. Louis, MO, USA), which inhibits phosphatidylinositol 3-kinas (PI3K) and then blocks the initial stage of autophagosome formation. CQ was purchased from Cell Signaling Technology (USA) and CQ blocks LC3-II degradation, thereby inhibit the later stage of autophagosome fusion. 3-MA or CQ was exposed to cells for 48 h.

### 2.2. Western Blot

The protein level of LC3 (I: 16kDa and II: 14 kDa, Cell Signaling Technology, USA), Beclin1 (52kDa, abcam, England), P62 (62kDa, abcam, England), and GAPDH (36kDa, Proteintech Group, Wuhan, China) were measured using a Western Blot detection system (GeneGnome 5, Cambridge, UK). Cells (2x10^5^) were incubated in 6-wells plates. Every plate was treated with different doses of TNF-*α* in the presence or absence of 3-MA (5 mM) for 48 h. The protein concentration was measured using the BCA method (Biossci, Wuhan,China), and samples were separated on a 5–12% sodium dodecyl sulfate polyacrylamide gel and transferred to a polyvinylidene fluoride membrane (Beyotime Biotechnology, Shanghai, China). These membranes were blocked in 5 % skim milk at room temperature for 1 h and incubated overnight at 4°C with the primary antibodies. Then membranes were washed and incubated with horseradish peroxidase-conjugated secondary antibody (Earthox, USA) at room temperature for 1 h. Band density was detected by image capture densitometry.

### 2.3. mRFP-GFP-LC3 Adenoviral Transfection and Confocal Microscopy

mRFP-GFP-LC3 adenoviral was purchased from HanBio Technology (Shanghai, China). Cells were cultured in 6-well plates and adenoviral transfection was performed when cells reached 50% confluence. Cells were incubated in cultured medium with the adenoviruses at a MOI of 100 for 36h at 37°C before the treatment of drugs. Autophagy was observed under a confocal microscopy (Olympus, Tokyo, Japan). Autophagic flux was detected by counting the number of red and yellow puncta (puncta/cell were counted). Red puncta were represented as autolysosome and yellow puncta were represented as autophagosome. Both of them represent the level of autophagy.

### 2.4. Flow Cytometry

Cells were harvested and washed with phosphate buffered saline (PBS) and resuspended in annexin V binding buffer (BD Biosciences, USA). Cells were incubated with fluorescein isothiocyanate–annexin V (BD Biosciences, USA) for 15 minutes at room temperature in the dark and propidium iodide (BD Biosciences, USA) for 5 minutes at 4°C in the dark. Next, cells were analyzed to flow cytometry (BD Biosciences, USA).

### 2.5. Statistical Analysis

Mean ±standard deviation (SD) values or fold changes were calculated. When subjected to normal distribution, the data were analyzed with two factors analysis of variance (two-way ANOVA), by using GraphPad Prism 5.0. P Value less than 0.05 was considered significant.

## 3. Results

### 3.1. TNF-*α* Induced Autophagosome Formation

**Figure 1:**
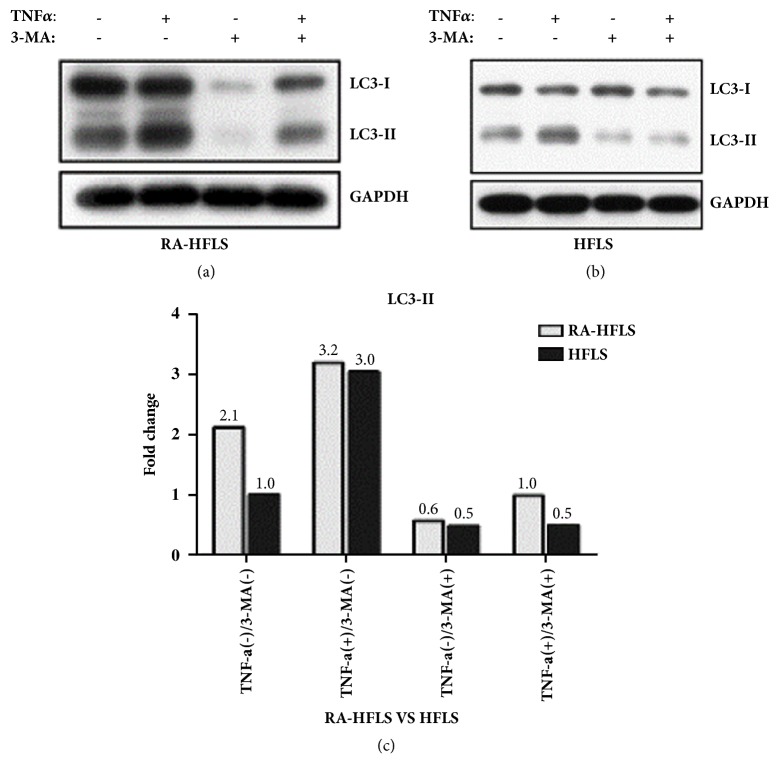
LC3 has two subtypes, LC3-I and LC3-II. When autophagy was induced, the conversion from LC3-I to LC3-II was correspondingly increased. LC3-II was commonly used as a maker for autophagosomes. RA-HFLS (a) and HFLS (b) were treated with 30 ng/ml TNF-*α* in presence or absence of 5 mM 3-methyladenine (3-MA) for 48 h. LC3 was detected by western blot, and GAPDH was used as a loading control in all experiments. Values are fold change, divided by the average value of relative optical density of LC3-II in the HFLS without any treatment (c).

Autophagy related protein type II Light Chain 3 (LC3-II) activation is a marker of autophagy, which was detected by Western Blot (Figure 1). According to Western Blot data, the basic level of autophagy in the RA-HFLS group was 2.1-fold as many as HFLS group. When RA-HFLS and HFLS were treated with TNF-*α* at concentration of 30ng/ml for 48h, autophagy was increased both in RA-HFLS group and HFLS group (3.2 fold, 3.0 fold), and HFLS group has a large range of increment compared to the RA-HFLS group. We also showed that 3-MA treatment inhibited the autophagic flux in both groups.

**Figure 2:**
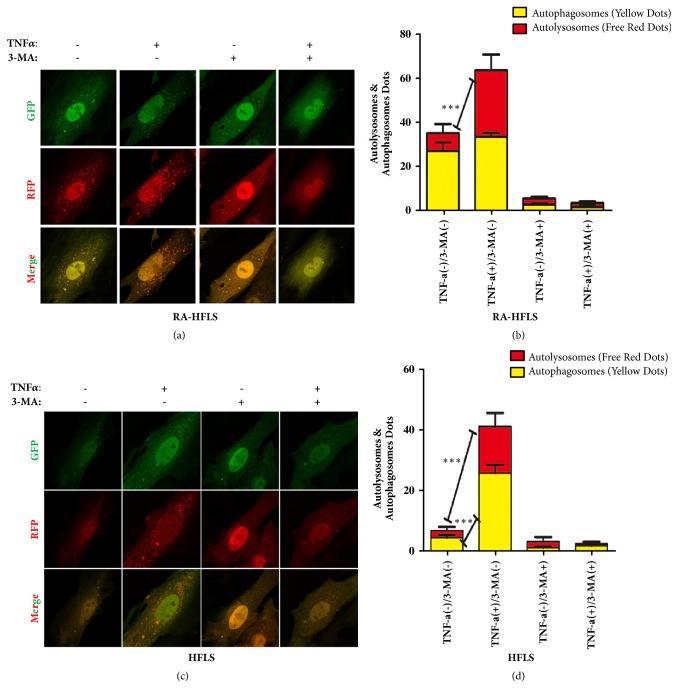
Cells were treated with 30ng/ml TNF-*α* in presence or absence of 5 mM 3-MA for 48 h. Cells were incubated in cultured medium with the mRFP-GFP-LC3 adenoviruses at a MOI of 100 for 36h at 37°C before the treatment with TNF-*α* and 3-MA. We counted yellow spots and red spots under the confocal microscope (a, c). The red spot represents autolysosome and yellow spot represents autophagosome. The percentage of yellow staining cells and red staining cells were shown in the bar graphs to evaluate autophagosome formation in each group (b, d). Values are mean± standard deviation of three independent experiments. *∗*p < 0.05, *∗∗*p < 0.01, and *∗∗∗*p < 0.001.

**Figure 3:**
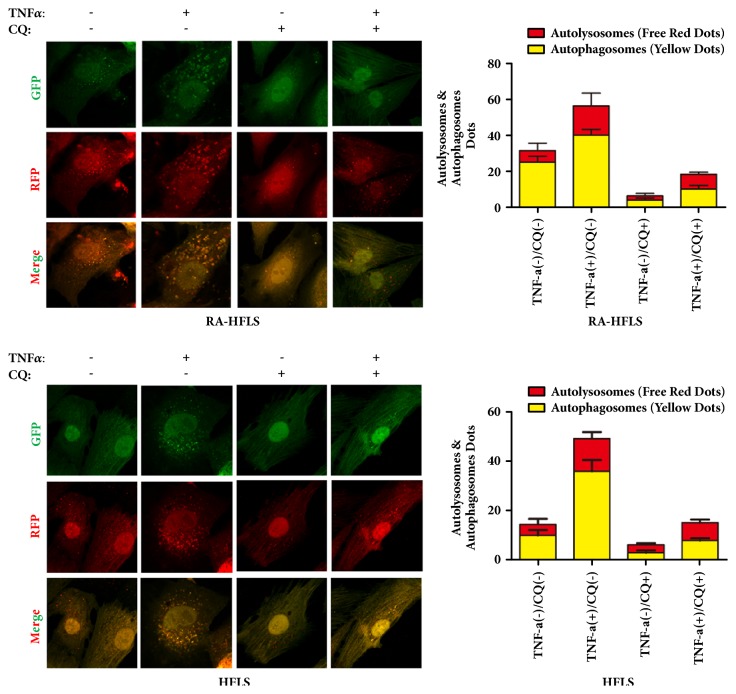
Cells were treated with 30ng/ml TNF-*α* in presence or absence of 30 uM CQ for 48 h. Cells were incubated in cultured medium with the mRFP-GFP-LC3 adenoviruses at a MOI of 100 for 36h at 37°C before the treatment with TNF-*α* and CQ. We counted yellow spots and red spots under the confocal microscope. The red spot represents autolysosome and yellow spot represents autophagosome. The percentage of yellow staining cells and red staining cells were shown in the bar graphs to evaluate autophagosome formation in each group. Values are mean± standard deviation of three independent experiments.

To further prove the mentioned result, we detected LC3-II in both group under the confocal microscopy. RA-HFLS and HFLS were transfected the mRFP-GFP-LC3 adenoviral first and then cultivated in the presence or absence of autophagy inhibitor 3-MA or CQ. We counted yellow spots and red spots under the confocal microscopy (Figures 2 and 3). These results also proved that RA-HFLS group has a higher the basic level of autophagy than HFLS. TNF-*α* induced autophagosome formation in both groups. RA-HFLS group have a higher autophagy level than in HFLS group under the treatment of TNF-*α*. 3-MA and CQ could inhibit autophagy in both group, which were consistent with the Western Blot results.

### 3.2. The Dose-Dependent Effect of TNF-*α* Enhanced Autophagy Was Not Found in RA-HFLS but in HFLS

**Figure 4:**
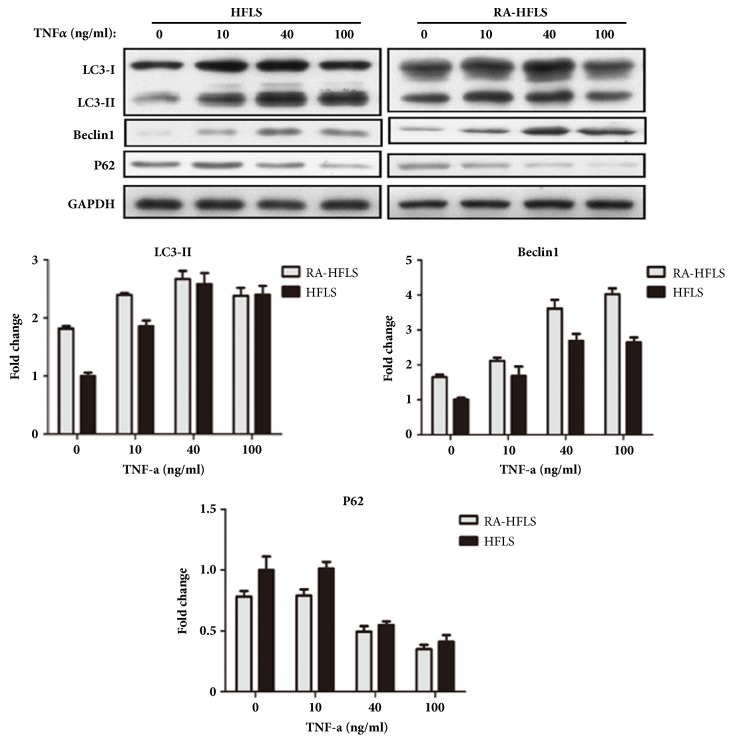
Cells were treated with TNF-*α* at concentrations 0, 10, 40, and 100 ng/ml for 48 h. LC3-II, Beclin1, and P62 were detected by western blot. Values are fold change, divided by the average value of relative optical density of these protein in the HFLS without any treatment. The data were analyzed and presented by histogram.

Both RA-HFLS and HFLS were treated with TNF-*α* at concentrations 0, 10, 40, and 100 ng/ml for 48 h. The level of LC3-II and Beclin1 increased in both group after the treatment with TNF-*α*; correspondingly, the level of P62 decreased in both group (Figure 4). According to these data, the autophagic flux was much higher in RA-HFLS than HFLS. When TNF-*α* was less than the concentration of 40 ng/ml, the level of autophagy increased in both groups. However, when TNF-*α* was at the concentration of 100 ng/ml, TNF-*α* no longer had a greater impact on inducing autophagy in both groups. The increasing of autophagy showed a TNF-*α* dose-dependent manner in HFLS (r2=0.457, P<0.05), but not in RA-HFLS (r2=0.161, P>0.05). Also, a bigger increasing range of autophagic flux was observed in HFLS group than in RA-HFLS group.

### 3.3. Autophagy Induction Increased the Resistance to TNF-*α* Induced Apoptosis

**Figure 5:**
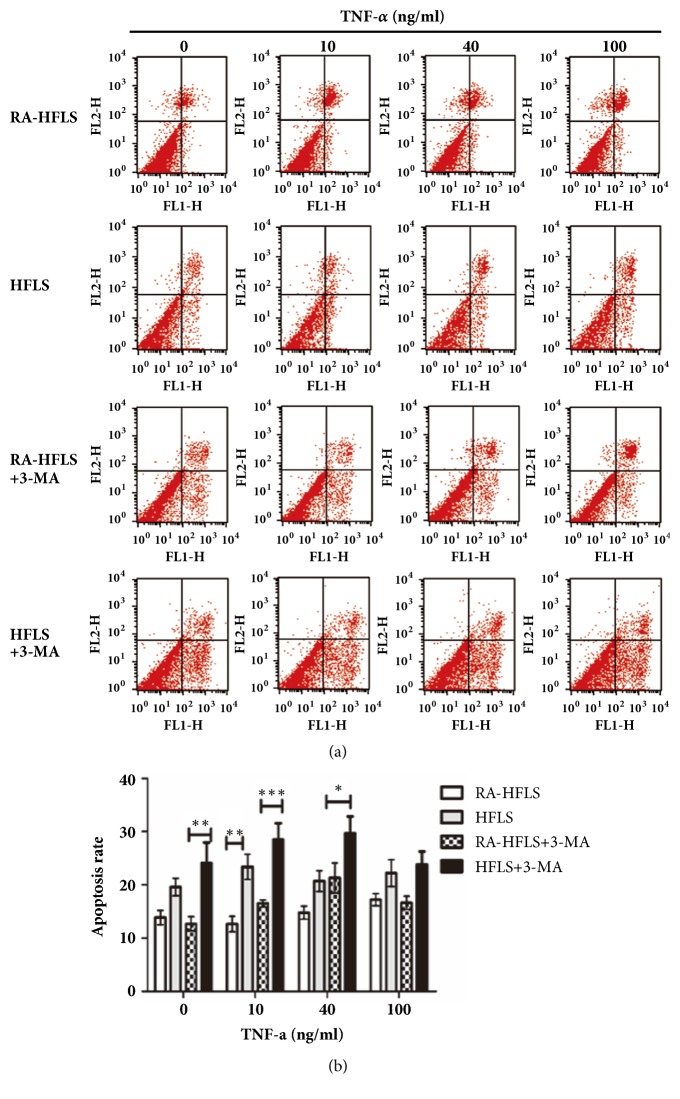
Cells were treated with 0, 10, 40, and 100ng/ml TNF-*α* in presence or absence of 5 mM 3-MA for 48 h. Apoptosis was determined by flow cytometry following annexin V/propidium iodide (PI) staining (a). The data were analyzed and presented by histogram (b). Values were mean± standard deviation. The two factors analysis of variance (two-way ANOVA) was chosen for analysis of data from cell apoptosis results. *∗*p < 0.05, *∗∗*p < 0.01, and *∗∗∗*p < 0.001.

TNF-*α* was reported to activate extrinsic apoptosis and contribute to programmed cell death [17]. We showed here that RA-HFLS and HFLS were exposed to TNF-*α* at concentrations 0, 10, 40, and 100 ng/ml for 48 h (Figure 5). With the treatment of TNF-*α*, apoptosis was induced in both groups but did not show a dose-development manner (p>0.05). Also, we found that RA-HFLS (16.51 ± 1.60 %) were more resistant to TNF-*α* (10 ng/ml) -induced apoptosis compared to HFLS (23.38± 5.83 %), with a significant difference (p<0.05). When RA-HFLS and HFLS were treated with other doses of TNF-*α* (0, 40, and 100 ng/ml), the apoptosis was higher in HFLS than RA-HFLS without a statistical significance (P>0.05). When RA-HFLS and HFLS were treated with different doses of TNF-*α* in the presence of the autophagy inhibitor 3-MA. It showed that TNF-*α*-induced apoptosis was increased in these two kinds of cells after blocking the autophagic pathway. HFLS had higher apoptosis level than RA-HFLS when TNF-*α* was at concentration of 0, 10, amd 40 ng/ml, and with a statistical significance (P<0.05).

### 3.4. Autophagy Was Inversely Proportional to Apoptotic in HFLS

**Figure 6:**
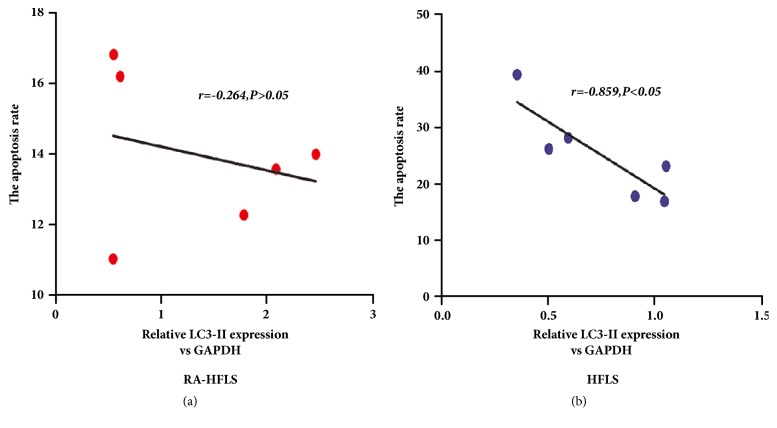
Cells were treated in the presence or absence of d 5 mM 3-MA for 48 h. Autophagy was detected by western blot (LC3-II) and cell apoptosis was determined by flow cytometry. The correlation between autophagy and apoptosis was analyzed with Pearson correlation in RA-HFLS (a) and in HFLS (b).

Increasing evidence indicated that RA-HFLS employed other survival strategy to inhibit apoptosis besides autophagy [18]. Therefore, we analyzed the correlations between autophagy and apoptosis in RA-HFLS and in HFLS with Pearson correlation (Figure 6). The data was analyzed including autophagy level and apoptosis level in both RA-HFLS and HFLS in the presence or absence of the autophagy inhibitor 3-MA. Autophagy showed the negative correlation of the level of apoptosis in HFLS (r=-0.859) with a statistical significance (P<0.05), but not in RA-HFLS (r=-0.264, p>0.05). We speculated that RA-HFLS might have other mechanisms to enhance the apoptosis-resistance besides the autophagy pathway.

## 4. Discussion

RA synovial cells can be divided into two subsets: fibroblast-like and macrophage-like synoviocytes. Fibroblast-like synoviocytes refer as to RA-HFLS here, which are primarily responsible for the inflammation and the destruction of articular cartilage and bone. However, the apoptosis resistance and proliferation of RA-HFLS seem to importantly resulting in synovial hyperplasia. Macrophage-like synoviocytes contribute to the production of proinflammatory cytokines, primarily TNF-*α*. Thereby macrophage-derived TNF-*α* increases the expansion of RA-HFLS [19, 20]. It is reported that TNF-*α* could induce autophagy in RA-HFLS and then increase the tolerance to apoptotic stimuli [18].

Autophagy has been shown to maintain organismal homeostasis by eliminating protein aggregates and damaged organelles. Recently it appears to a complex interplay with cell survival. When autophagy could not handle with those stimuli, it induces an autophagic cell death pathway [21]. Kato and colleagues demonstrated autophagy seemed to play a dual role in the survival of RA-HFLS. Autophagy could also lead to cell death in RA-HFLS in response to severe endoplasmic reticulum stress, so-called type 2 non-apoptotic cell death [22]. At most times, it functions as a protector in many cells by inhibiting apoptosis. Autophagy and apoptosis are not completely independent pathway, there is a complex intersection between them. Recent studies have showed that some molecular were involved in the crosstalk between autophagy and apoptosis, such as B-cell lymphoma-2 (Bcl-2) and Bcl-2-like protein (Bcl-xL). Bcl-2 and Bcl-xL were characterized as anti-apoptosis molecules. However, they appeared to inhibit autophagy by binding to Beclin1 [23, 24]. To clarify the consequences of autophagy and apoptosis induced by TNF-*α* in RA-HFLS, we detected autophagy and apoptosis in RA-HFLS and HFLS treated with different doses of TNF-*α*. Autophagy activity was increased by TNF-*α* in RA-HFLS, but not in a dose-dependent manner. Autophagy inhibitor 3-MA significantly decreased the autophagy and subsequently resulted in increased apoptosis with TNF-*α* treatment. Under our experimental conditions, TNF-*α* induced autophagy served as a protector in RA-HFLS.

It was reported that autophagy and proteasome degradation pathways were more active in RASFs compared with control fibroblasts in treatment of TNF-a [18]. However, the capacity of RA-HFLS for apoptotic resistance was rarely mentioned when it was exposed to different doses of TNF-a. In our study, enhanced LC3 and beclin1 expression were observed in RA-HFLS compared with the HFLS group without any stimulation, which means that RA-HFLS has higher basal autophagy than the HFLS group. Autophagy was increased both in RA-HFLS group and HFLS in the presence of TNF-a. However, HFLS group has a large range of increment than the RA-HFLS group. TNF-*α*-induced autophagy was observed in a dose-dependent effect in HFLS but not in RA-HFLS. This phenomenon might be a result of both autophagy and proteasome degradation pathways enhanced in RA-HFLS in the presence of TNF-a. We found that when RA-HFLS was treated with different doses of TNF-a in the presence of autophagy inhibitor 3-MA or CQ, apoptosis further accumulated. This finding also indicated enhancement of autophagic flux could increase the tolerance to apoptosis induced by TNF-a. In contrast to HFLS, less apoptosis level was observed in RA-HFLS treated with TNF-a in the presence or the absence of 3-MA. Apart from autophagy pathway, other protection mechanisms, liking proteasome degradation pathways, might keep RA-HFLS from apoptosis. Here, we need to observe the change that a single factor (TNF-a) induce the apoptosis of RA-HFLS in vitro experiment. Thus, the concentration of TNF-a used in our experiment were much higher than its concentration in RA patient's peripheral serum.

The results of the present study indicated that RA-HFLS might use the autophagic pathway as a survival mechanism in the condition of TNF-a stress, and TNF-a-induced autophagy was not in dose dependent manner. Thus, autophagy inhibitors might be a new treatment of RA.

## Data Availability

The data used to support the findings of this study are available from the corresponding author upon request.
